# Specificity in Mesograzer-Induced Defences in Seagrasses

**DOI:** 10.1371/journal.pone.0141219

**Published:** 2015-10-27

**Authors:** Begoña Martínez-Crego, Pedro Arteaga, Alexandra Ueber, Aschwin H. Engelen, Rui Santos, Markus Molis

**Affiliations:** 1 Centre of Marine Sciences (CCMAR), Faro, Portugal; 2 University of Rostock, Rostock, Germany; 3 Alfred-Wegener-Institut, Helmholtz-Zentrum für Polar- und Meeresforschung, Section Functional Ecology, Bremerhaven, Germany; The University of Hong Kong, HONG KONG

## Abstract

Grazing-induced plant defences that reduce palatability to herbivores are widespread in terrestrial plants and seaweeds, but they have not yet been reported in seagrasses. We investigated the ability of two seagrass species to induce defences in response to direct grazing by three associated mesograzers. Specifically, we conducted feeding-assayed induction experiments to examine how mesograzer-specific grazing impact affects seagrass induction of defences within the context of the optimal defence theory. We found that the amphipod *Gammarus insensibilis* and the isopod *Idotea chelipes* exerted a low-intensity grazing on older blades of the seagrass *Cymodocea nodosa*, which reflects a weak grazing impact that may explain the lack of inducible defences. The isopod *Synischia hectica* exerted the strongest grazing impact on *C*. *nodosa* via high-intensity feeding on young blades with a higher fitness value. This isopod grazing induced defences in *C*. *nodosa* as indicated by a consistently lower consumption of blades previously grazed for 5, 12 and 16 days. The lower consumption was maintained when offered tissues with no plant structure (agar-reconstituted food), but showing a reduced size of the previous grazing effect. This indicates that structural traits act in combination with chemical traits to reduce seagrass palatability to the isopod. Increase in total phenolics but not in C:N ratio and total nitrogen of grazed *C*. *nodosa* suggests chemical defences rather than a modified nutritional quality as primarily induced chemical traits. We detected no induction of defences in *Zostera noltei*, which showed the ability to replace moderate losses of young biomass to mesograzers via compensatory growth. Our study provides the first experimental evidence of induction of defences against meso-herbivory that reduce further consumption in seagrasses. It also emphasizes the relevance of grazer identity in determining the level of grazing impact triggering resistance and compensatory responses of different seagrass species.

## Introduction

Plants and herbivores are involved in complex interactions, in which plants play an active role. Terrestrial plants, freshwater macrophytes, and seaweeds may respond to herbivory by inducing plastic defences to prevent further attacks (reviewed in [1, 2, 3]). Inducible defences involve several structural and chemical traits that decrease plant palatability or attractiveness to herbivores with negative effects on their preference or fitness [4, 5, 6]. Structural phenotypic responses may involve rapid morphological changes such as spines and adventitious branching, as well as mechanical barriers such as toughened or hardened leaves, increased mineral content or cell wall thickness and lignification [7, 8]. Major known inducible chemical defences include a wide range of defensive proteins and secondary metabolites that have toxic, deterrent, and/or digestion-reducing effects on herbivores [9, 10]. Induced defences may have large effects on herbivore populations and community structure (e.g. [11, 12, 13]).

In spite of their widely recognized value, grazer-induced defences are not expressed by all primary producers or equally effective against all grazers. In the marine environment, for instance, brown and green, but not red, seaweeds induce chemical defences in response to grazing by small crustaceans and gastropods, but not in response to large gastropods and sea urchins (meta-analysis by [2]). According to the optimal defence theory (ODT), such inter-specific differences are expected because inducible defences are favoured if the grazing impact is high enough to reduce plant fitness [14]. Grazing impact is linked to herbivore size, life history and mobility, which also influence the grazer-specificity in the effectiveness of induced defences (e.g. [15, 16, 2]). Grazing impact depends not only to the amount of damage but also on the fitness value of the specific tissue of the plant consumed by the grazer. For instance, herbivores that selectively feed on young, metabolically active tissues are expected to have a much larger impact on plant fitness than herbivores that prefer older and senescent tissues [17, 7]. At the same time, plant tolerance mechanisms such as compensatory growth may reduce the impact of herbivores on plant fitness [18, 19] and preclude the induction of defences in some cases [20]. Lastly, effectiveness of inducible defences depends on their temporal progression, which should minimize the period of vulnerability experienced by plants between the damage and the induction [1].

Seagrasses are foundation species that form highly productive meadows, which provide valuable ecosystem services such as food and habitat to a large number of associated organisms, oxygen production, and sink for CO_2_ emissions [21, 22]. They posses a variety of secondary metabolites that may act as chemical defences [23, 24], and exhibit a high phenotypic plasticity in their physiological [25, 26], structural and biomechanical [27, 28] responses to environmental stressors. We therefore expect seagrasses to induce defences against herbivory, a fact that to our knowledge has not been demonstrated so far. Compared to the extensive knowledge on terrestrial plants and seaweeds, our understanding of the grazer-induced defences in seagrasses is scarce. The few studies conducted on seagrasses have shown no induction of anti-herbivory defences in response to direct grazing by macrograzers [29] or to simulated herbivory [30, 31]. However, small herbivores (mesograzers) rather than macrograzers or simulated herbivory are expected to trigger the induction of anti-herbivory defences. Mesograzers have reduced home ranges and inflict a gradual removal of selected tissues over a prolonged period, whereas macrograzers can consume entire plant individuals in a short feeding attack and do not stay on the same plant long enough to suffer the induced response [32]. Similarly, grazer-damage has been reported to induce a different plant response than physical damage alone [33, 34, 35]. Despite these indications, in a recent study, Steele and Valentine [36] found no induced change in the palatability of two tropical seagrass species to the isopod *Paracerceis caudata* after a 15-day induction experiment. Therefore, in order to understand the general significance of inducible defences in seagrasses we first need to provide a solid assessment of whether mesograzers are able to induce defences that deter consumption and how this may vary among different seagrass and mesograzer species.

In this study, we investigated the ability of two NE Atlantic seagrass species, *Zostera noltei* and *Cymodocea nodosa*, to induce defences against direct grazing by different mesograzer species that use these seagrasses as both, habitat and food. Specifically, we used feeding-assayed induction experiments to examine seagrass and mesograzer specificity in the induction of defences within the context of the ODT predictions by asking: (1) do higher grazing intensity and the selective feeding on young tissues with higher fitness value favour the induction of defences? (2) does compensatory growth preclude the induction of defences? and if induction is detected (3) what is its nature (chemical or structural) and its temporal progression?

## Material and Methods

### Study site and organisms

**Fig 1:**
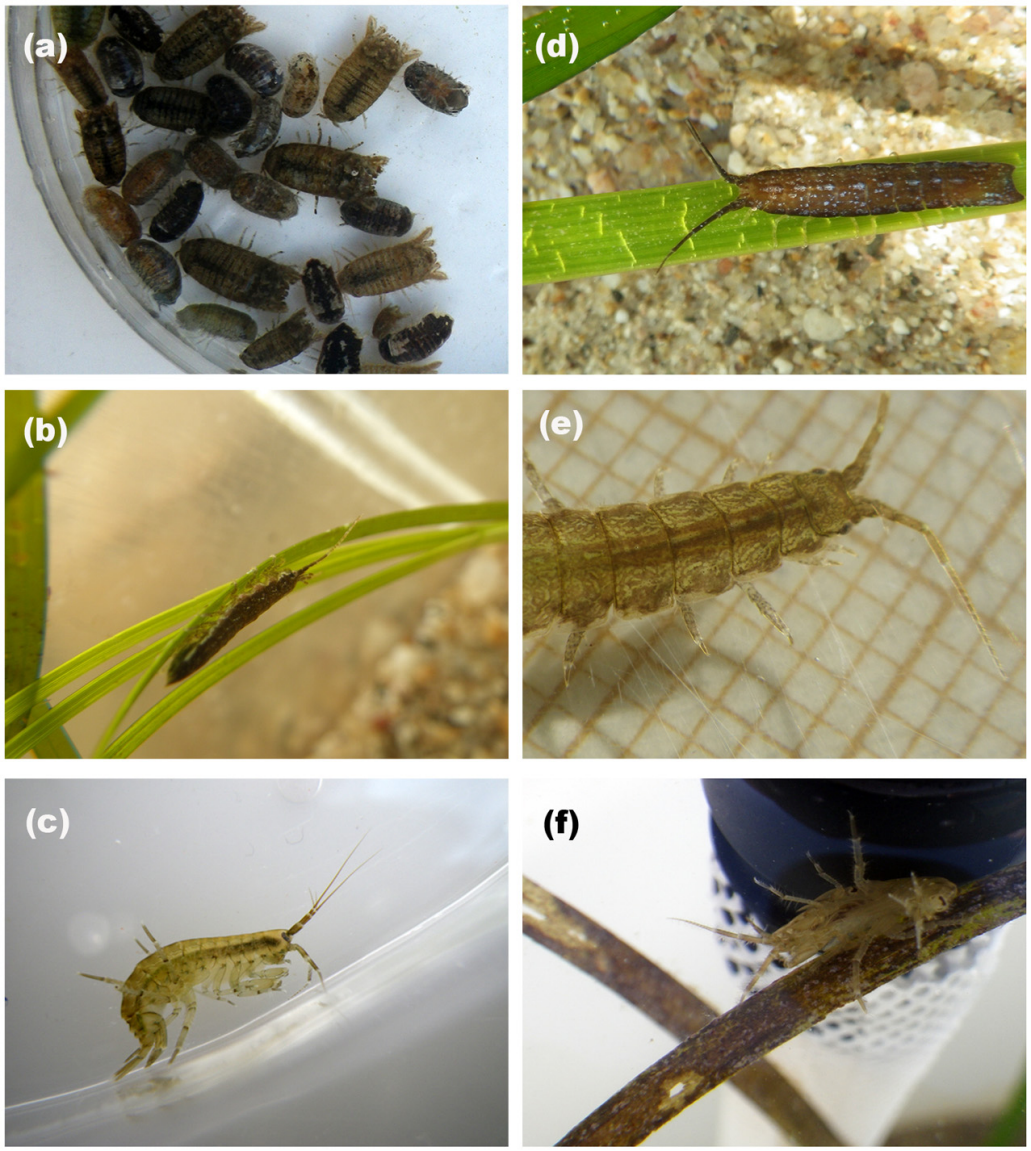
Study organisms. *Zostera noltei* shoots were exposed to grazing by *Cymodoce truncata* (a), *Idotea chelipes* (b), and *Gammarus insensibilis* (c), and *Cymodocea nodosa* shoots were exposed to grazing by *Synischia hectica* (d), *Idotea chelipes* (e), and *Gammarus insensibilis* (f).

Two seagrass species and four of their associated mesograzer species were collected within the Ria Formosa lagoon (37°00´N, 7°53´W, NE Atlantic, Southern Portugal). This shallow mesotidal lagoon is located on a wave-sheltered shore with semidiurnal tides having a range of 3 m. Dense *Z*. *noltei* meadows dominate the intertidal [37], while *C*. *nodosa* meadows are located at the subtidal [38]. Seagrass shoot-specific biomass (mean ± SE) measured in the study site at the moment of plant collection was 0.03 ± 0.002 g dry weight for *Zostera noltei* (n = 13) and 0.17 ± 0.01 g dry weight for *Cymodocea nodosa* (n = 15). The study encompassed direct grazing by *Idotea chelipes* (1.3 ± 0.03 cm length), *Cymodoce truncata* (1.0 ± 0.03 cm), and *Gammarus insensibilis* (1.7 ± 0.04 cm) on *Zostera noltei*, and by *Synischia hectica* (3.2 ± 0.1 cm), *I*. *chelipes* (1.5 ± 0.03 cm), and *G*. *insensibilis* (1.8 ± 0.04 cm) on *Cymodocea nodosa* (Fig 1). At the study site, the amphipod *G*. *insensibilis* and the isopods *I*. *chelipes* and *C*. *truncata* are associated with both seagrass species, while the isopod *S*. *hectica* is solely associated with larger seagrasses like *C*. *nodosa*. Based on previous tests, *C*. *truncata* feeding on *C*. *nodosa* was rare. Ria Formosa is a Natural Park and permission for sampling was provided by the Portuguese ICNF (Instituto da Conservação da Natureza e das Florestas). No protected species were sampled.

### Induction phase

To investigate whether direct grazing by mesograzers induces anti-herbivory defences in seagrasses, induction experiments with a total of six different seagrass-grazer combinations (see above and Fig 1) were conducted at the Ramalhete field station (CCMAR) during May-June 2014. Nine replicates were randomly assigned to each combination, except for the grazers with the highest consumption on each seagrass species based on previous tests (i.e. *Z*. *noltei*-*I*. *chelipes* and *C*. *nodosa*-*S*. *hectica*), which were replicated 12 times. Each replicate consisted of one treatment and one control container, which were arranged in pairs to maximise similarity in their experimental conditions. In each container 6 seagrass shoots lacking visual feeding scars were planted into sieved and cleaned sand, after carefully removing most macroscopic epibionts from all blades with a glass slide to avoid confounding effects of co-consumption [39]. *Z*. *noltei* shoots were planted in containers of 2.2 L (168 x 115 x 115 mm) allocated outdoor and individually supplied with seawater at a mean (± SE) flow rate of 215 (± 18) mL min^-1^. The mean (± SE) seawater temperature in these containers during the experiment was 22.1 (± 0.05°C (HOBO dataloggers). *C*. *nodosa* shoots were planted in cylindrical containers of 26 L (272 mm in diameter and 455 mm in height) also allocated outdoor and individually supplied with seawater at a fixed flow rate of 1000 mL min^-1^. Within these containers, mean (± SE) seawater temperature was 22.2 (± 0.03°C. The mean (± SE) ambient salinity (VWR portable conductivity meter) and irradiance at the blade height (PAR Odyssey-Dataflow Systems Pty Ltd.) during the experiment were 36.3 ± 0.1 ‰ and 325 (± 14) μmol m^-2^ s^-1^, respectively. To avoid animal escapes, the effluent pipe was fixed in each container below the upper margin and covered with a polypropylene net of ca. 1 mm mesh size. Epibionts were biweekly removed from container walls using a sponge and from seagrass blades by carefully gliding two fingers along each leaf to avoid any abrasion.

Plants were left to acclimatize for 5 days in order to allow recovery from transplantation stress and to reduce any potential defensive trait attained by unknown grazing histories in the field. Afterwards, the induction phase started by adding 2 individuals of a grazer species (inducers) to each of the treatment containers of each seagrass-grazer combination, while no grazer was added to the control containers. After 5, 12, and 16 days of the induction phase, two seagrass shoots were removed from each control and treatment container. From these shoots, blades were selected for feeding assays with fresh food or selected and frozen in liquid nitrogen for agar-reconstituted food (see details below). The induction phase for *I*. *chelipes*-exposed *C*. *nodosa* was extended from 12 to 20 days, because this isopod species needed more time to start grazing on *C*. *nodosa* shoots (almost no bite marks present after 10 days; see results). One grazer was removed from each treatment container after the removal of the first pair of shoots to apply a comparable grazing pressure to the remaining seagrass shoots during the induction phase.

Grazing impact during the induction phase was monitored ca. every 3 days by recording the number and position of new feeding marks on each blade within a shoot. Bite position was related to tissue age by dividing each blade into basal, middle, and apical sections and assigning each section to an age category. These categories were defined based on seagrass growth pattern (i.e. youngest leaf located in the innermost part within the shoot and young tissues growing from the meristem in the leaf base upwards with older tissues being brownish and not actively growing) as in Casola et al. [40]. Categories for the study species and season were as follows: Young = full youngest (innermost) leaf, basal and middle sections of second innermost leaf, and basal part of third leaf; Intermediate = apical part of second leaf, middle section of third leaf, and basal part of fourth leaf; Old = apical section of third leaf, middle and apical parts of the fourth and fifth leaves. We conducted a repeated measures analysis of variance (RM-ANOVA) to assess the effects of time (within-subject measure, four levels) and grazer species (between-subject factor, three levels) on the grazing impact estimated as the proportion of grazed blades. This response variable considered the total number of leaves per shoot, including newly produced and lost older leaves. In addition, we performed a RM-ANOVA to assess the effects of the age of the blade (within-subject measure, three levels: young, intermediate, and old) and grazer species (between-subject factor, three levels) on the grazing impact estimated as the total number of bite marks accumulated during the induction phase. The age of the consumed blades was used as indicator of the grazing impact on plant fitness. Tissue age was a dependent measure (within-subject) because young, intermediate and old blades were simultaneously exposed to grazing in each container. The mean value per container of each response variable was used as replicate and data for each seagrass species were analysed separately. Significant effects and interactions in the two-way RM-ANOVAs were further investigated using one-way RM-ANOVAs conducted for each grazer separately. As post-hoc tests, we used paired t tests or non-parametric Wilcoxon signed-ranks paired tests when normality assumption was not meet even after trying several transformations. As the probability of finding the obtained number of significant one-way RM-ANOVA or t tests by chance was < 5%, no Bonferroni correction was calculated [41]. When data did not meet sphericity (Mauchly´s test) corrected degrees of freedom from Greenhouse-Geisser adjustment were used. When data were non-normal or showed unequal variances associated to between-subject factors, the large sample size used led us to consider analyses of variance robust enough to allow departures from these assumptions [42].

Based on the results from the first induction experiment, the *Z*. *noltei*-*I*. *chelipes* and *C*. *nodosa*-*S*. *hectica* combinations were used in a second experiment to assess the potential deployment of seagrass compensatory responses during the induction phase. The experimental setup and conditions, as well as results of feeding assays with fresh seagrass, were similar to those of the first induction experiment but with a reduced number of shoots (2) in each replicate container (n = 12) and no time replication. Particularly, we measured shoot-specific leaf growth rate and examined its influence on the differences in leaf biomass between grazed and ungrazed shoots. Leaf growth was measured by punching with a needle all seagrass leaves within a shoot just above the sheath of the outermost leaf at the beginning of the induction phase. Eight days later punched shoots were collected and leaf growth was determined following a method modified from Zieman [43]. The leaf biomass formed after the punching in each marked shoot was measured and weighted and shoot-specific growth rates were expressed in g FW shoot^-1^ day^-1^. Leaf biomass was also dried to constant weight at 60°C and weighted to obtain values of shoot biomass. Again, the mean value per container of each response variable was used as replicate and data for each seagrass species were analysed separately. Significant differences between grazed and ungrazed shoots were investigated using unpaired t tests after checking normality and equal variances.

### Feeding assays

To test whether previous grazing by conspecific mesograzers changes the palatability of fresh seagrass blades, two-choice feeding assays were conducted at 3 times during the induction phase. In each replicate assay we offered a choice between one previously ungrazed and one previously grazed blade, corresponding to one replicate of the induction experiment. Selected grazed and ungrazed blades were of similar size and of the same tissue age consumed during the induction phase by each mesograzer species. Each replicate assay consisted of two feeding arenas (plastic aquaria of 1 L), one containing seagrass blades exposed to one naïve conspecific grazer and the other containing seagrass blades with no grazer to correct consumption for non-feeding related (autogenic) changes in blade wet masses during assays. Both arenas were arranged in pairs to maximise similarity in their experimental conditions. Prior to the start of the assays, grazers were acclimatized and fed fresh *Ulva* spp. for 24 hours in order to standardise their feeding history.

Feeding assays were run indoor, where fluorescent tubes irradiated feeding arenas with 10.6 ± 0.02 μmol m^-2^ s^-1^ (mean ± SE) PAR in a 13:11h light-dark cycle that matched the natural cycle. Mean (± SE) seawater temperature and salinity in feeding arenas were 19.2 (± 0.03°C and 36.6 (± 0.1) ‰, respectively. Seawater in feeding arenas was exchanged every 12 h. Assays lasted 3 days or until roughly half of one blade was consumed, whichever came first. Consumption of each seagrass blade offered to a grazer was calculated as [(H_i_ x C_f_/C_i_)-H_f_], where H_i_ and H_f_ were initial and final wet masses of the offered blade and C_i_ and C_f_ were initial and final wet masses of the corresponding autogenic control [44].

More consumption of previously ungrazed than grazed blades indicated an induction of grazer-deterrent traits in the seagrass, which can be of chemical and/or structural nature. To investigate whether grazer-deterrent effects observed with fresh food were due to induced changes in chemical rather than structural seagrass traits, additional feeding assays with agar-reconstituted food were conducted following a procedure adapted from Hay et al. [45]. Frozen (-80°C) seagrass blades were freeze-dried, ground to a homogenous fine powder, and 0.1 g of this powder was suspended in 1 ml distilled water. This seagrass suspension was mixed with molten agar (0.1 g in 1.5 ml of boiling distilled water). To prevent thermal breakdown of bioactive chemical metabolites, the agar solution was allowed to cool to about 55°C before mixing. The seagrass-agar mixture was poured over a mosquito mesh, flattened between two glass panels to obtain a uniform thickness, and allowed to cool for 1 h at 19°C. The solidified mixture adhered to the mesh was cut into pellets of 15 x 18 mm. Pellets containing previously ungrazed and grazed blades were offered to a single grazer in identical conditions to those of assays with fresh material. Consumption of each choice was calculated as described above for the fresh food.

A three-way RM-ANOVA was used for each seagrass species to test the effects of previous grazing (within-subject measure, two levels: ungrazed and grazed) on seagrass consumption by the different grazer species (between-subject factor, three levels) after different times of previous grazing (between-subject factor, three levels). Grazing was the dependent measure (within-subject) because previously ungrazed and grazed blades were simultaneously offered to one grazer in feeding arenas. Measurements of consumption at different times (between-subject factor) were independent because separate feeding assays with blades from different shoots were conducted at different times. As the within-subject factor had only two levels, testing for sphericity was not applicable. Significant interactions were further investigated using two-way RM-ANOVAs conducted for each grazer separately. When a significant effect of the factor grazing was detected in assays using fresh and reconstituted food, the effect sizes for each type of assay were compared in order to test any combined deterrent effect of structural traits in addition to chemical traits (i.e. a lower effect size with agar-reconstituted food than with fresh seagrass). Effect sizes were separately calculated for each type of assay as the difference between consumption of grazed and ungrazed material and compared using a non-parametric Mann-Whitney test because data were not normally distributed even after trying several transformations.

Total consumption rate in each feeding assay with fresh blades was also used to estimate the equivalent proportion of the shoot-specific leaf biomass (measured at the beginning of the induction experiments) that would be consumed on average by each grazer species in 8 days as indicator of the mesograzer-specific grazing intensity. The proportion was estimated for 8 days of consumption to allow a direct comparison with the loss of biomass measured in the growth induction experiments (data from 2 of the 6 seagrass-grazer combinations).

### Seagrass chemical traits

When an induction of chemical defences was suggested in feeding assays with agar-reconstituted food, we quantified seagrass nutritional value (C:N ratio and total nitrogen) and secondary compounds (total phenolics) to determine whether variation in those traits was induced by grazing. To obtain enough material for chemical analyses, remaining seagrass powder from assays with agar reconstitute food was pooled into two replicates of grazed and two replicates of ungrazed samples. Carbon and nitrogen content were analysed using a Carlo-Erba elemental analyser. Total phenolics were extracted with methanol and determined with spectrophotometer following a modified Folin-Ciocalteu assay using caffeic acid as standard (modified from Bolser et al. [46]). Difference between grazed and ungrazed material were tested for each chemical trait using Welch´s t tests, which are robust against the unequal variances detected for all variables.

## Results

### Grazing impact during the induction phase

**Fig 2:**
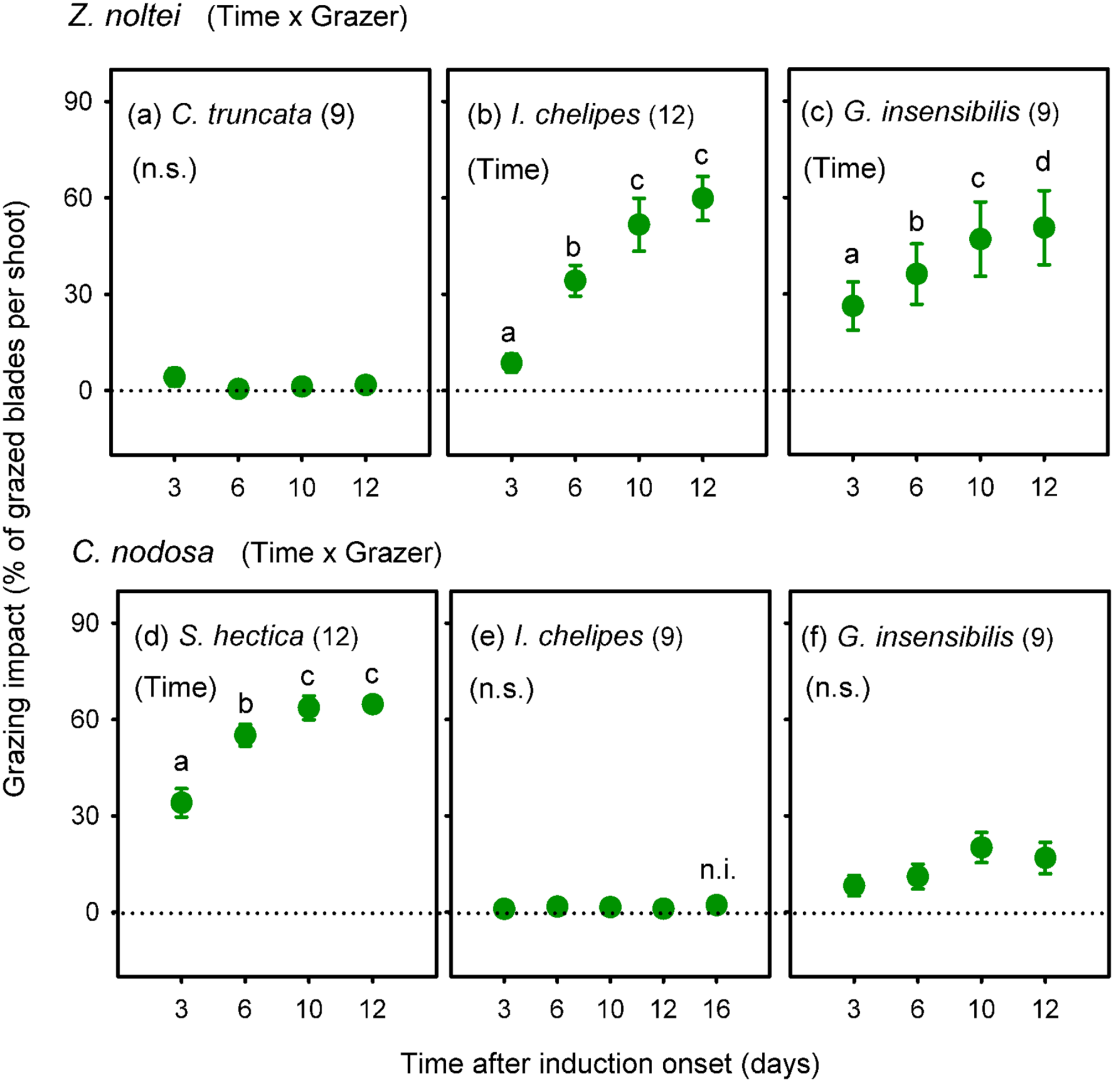
Grazing impact through time during the induction phase. Mean (± SE) proportion of grazed blades per shoot of the seagrass *Zostera noltei* (a-c) and *Cymodocea nodosa* (d-f) after 3, 6, 10, 12 or 16 days of exposure to different mesograzer species. Significant interaction for each seagrass species separately is shown in parentheses following the seagrass name. Significant Time effect for each grazer separately is shown in parentheses within each graph (n.s. = non-significant). Different letters above points in time denote statistically significant differences in grazing impact. Numbers in parentheses indicate sample size at each time (n.i. = data not included in the statistical analyses).

Grazing on *Z*. *noltei* by the isopod *I*. *chelipes* and the amphipod *G*. *insensibilis* was similar and significantly higher, most of the time, than the negligible grazing by *C*. *truncata* (significant Grazer x Time interaction in Fig 2a–2c and S1 Table). *I*. *chelipes* and *G*. *insensibilis* grazed more than 50% of *Z*. *noltei* blades after 12 days, but only grazing by *I*. *chelipes* showed an asymptotic attenuation that reflected a reduction in the grazing impact in the last time interval (Fig 2b and 2c).

Grazing impact on *C*. *nodosa* by the isopod *S*. *hectica* was significantly higher than by *G*. *insensibilis* and by *I*. *chelipes*, with the latter being negligible and significantly lower than by *G*. *insensibilis* at some times (significant Grazer x Time interaction in Fig 2d–2f and S1 Table). The temporal progression of the grazing impact exerted by *S*. *hectica* on *C*. *nodosa* reflected an asymptotic attenuation starting after 6 days and attaining a final value of more than 60% of *C*. *nodosa* blades after 10 days that was maintained after 12 days (Fig 2d). Grazing impact by the amphipod *G*. *insensibilis* was relatively low and irregular during the induction phase averaging 14.1% of grazed blades (Fig 2f).

**Fig 3:**
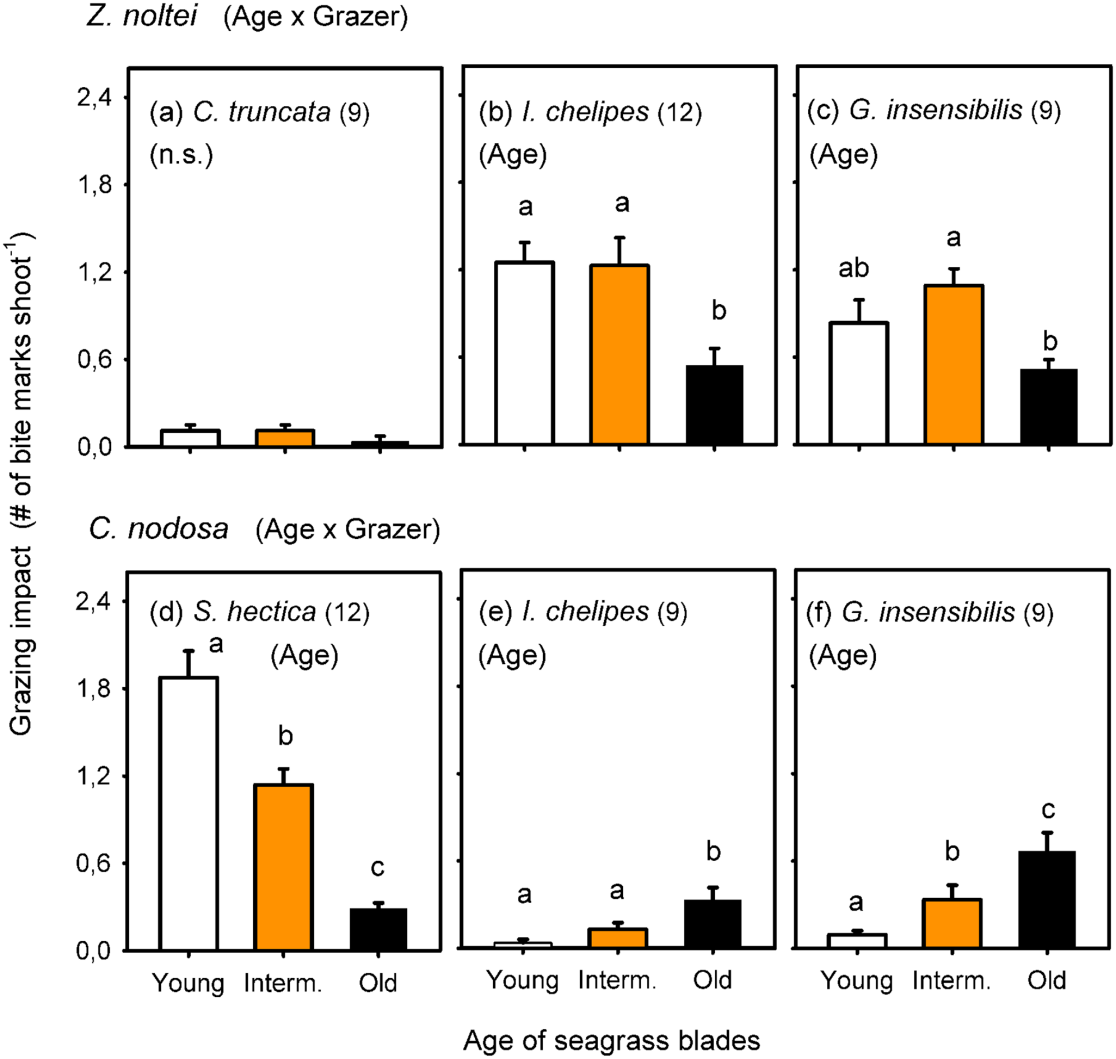
Grazing impact on seagrass blades of different age during the induction phase. Mean (± SE) number of bite marks by different mesograzer species in blades of different age of *Zostera noltei* (a-c) and *Cymodocea nodosa* (d-f). Significant interaction detected for each seagrass species separately is shown in parentheses following the seagrass name. Significant Age effect for each grazer separately is shown in parentheses within each graph (n.s. = non-significant). Different letters above age bars denote statistically significant differences in grazing impact. Numbers in parentheses indicate sample size.

Grazing by all mesograzers differently affected seagrass blades of different age, with the exception of *C*. *truncata* grazing on *Z*. *noltei* (Fig 3a and S2 Table). *I*. *chelipes* and *G*. *insensibilis* fed preferentially on intermediate and/or young blades of *Z*. *noltei* (Fig 3b and 3c), but preferred old tissues of *C*. *nodosa* (Fig 3e and 3f). *S*. *hectica* fed preferentially on young blades of *C*. *nodosa* (Fig 3d). The overall grazing impact in *Z*. *noltei* and *C*. *nodosa* blades of all ages (Fig 3a–3f) further confirmed the pattern of differences between grazers on the proportion of grazed blades (Fig 2a–2f).

### Seagrass compensatory response in the induction phase

**Fig 4:**
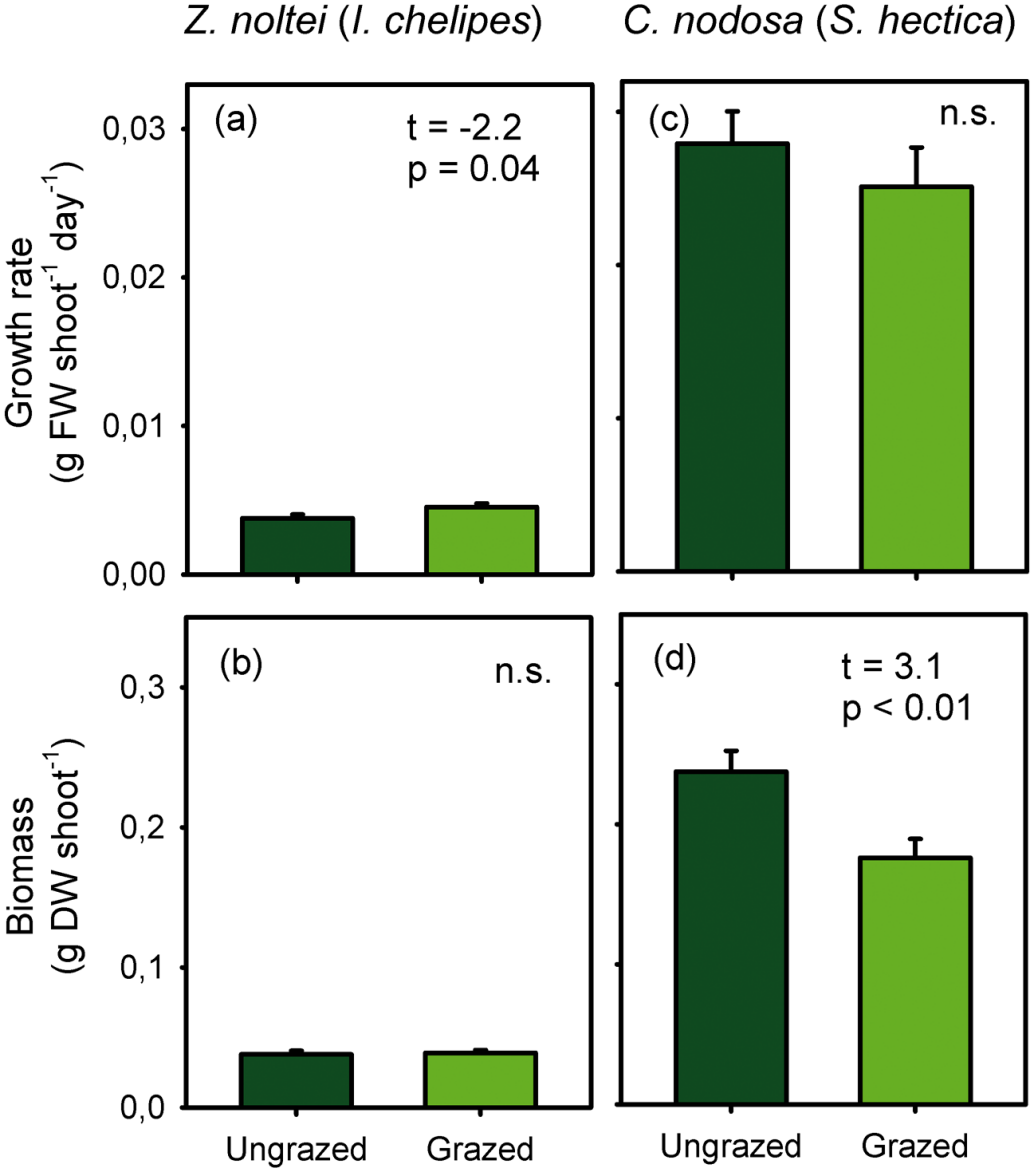
Shoot-specific leaf growth rate and biomass in the induction phase. Mean (± SE) values of *Zostera noltei* exposed to grazing by *I*. *chelipes* (a-b) and *Cymodocea nodosa* exposed to grazing by *S*. *hectica* (c-d). Statistics of unpaired t tests showing significant differences between grazed and ungrazed shoots are shown (n = 12; n.s. = non-significant). FW = fresh weight, DW = dry weight.

Leaf growth rate significantly increased in *Z*. *noltei* shoots exposed to direct grazing by the isopod *I*. *chelipes* (Fig 4a and S3 Table). This compensatory growth resulted in the lack of detection of biomass loss in grazed *Z*. *noltei* shoots (Fig 4b). In contrast, no increase in growth rate was detected in *C*. *nodosa* shoots exposed to *S*. *hectica* grazing (Fig 4c), which showed a significant decrease in leaf biomass (Fig 4d).

*C*. *nodosa* showed a faster growth rate than *Z*. *noltei* (Fig 4a and 4c), which was closely related to its higher shoot size as reflected by their similar growth rates of ungrazed shoots relative to natural shoot biomass (i.e. 35 and 30 days for total shoot biomass regrowth in *Z*. *noltei* and *C*. *nodosa*, respectively).

### Feeding assays

**Fig 5:**
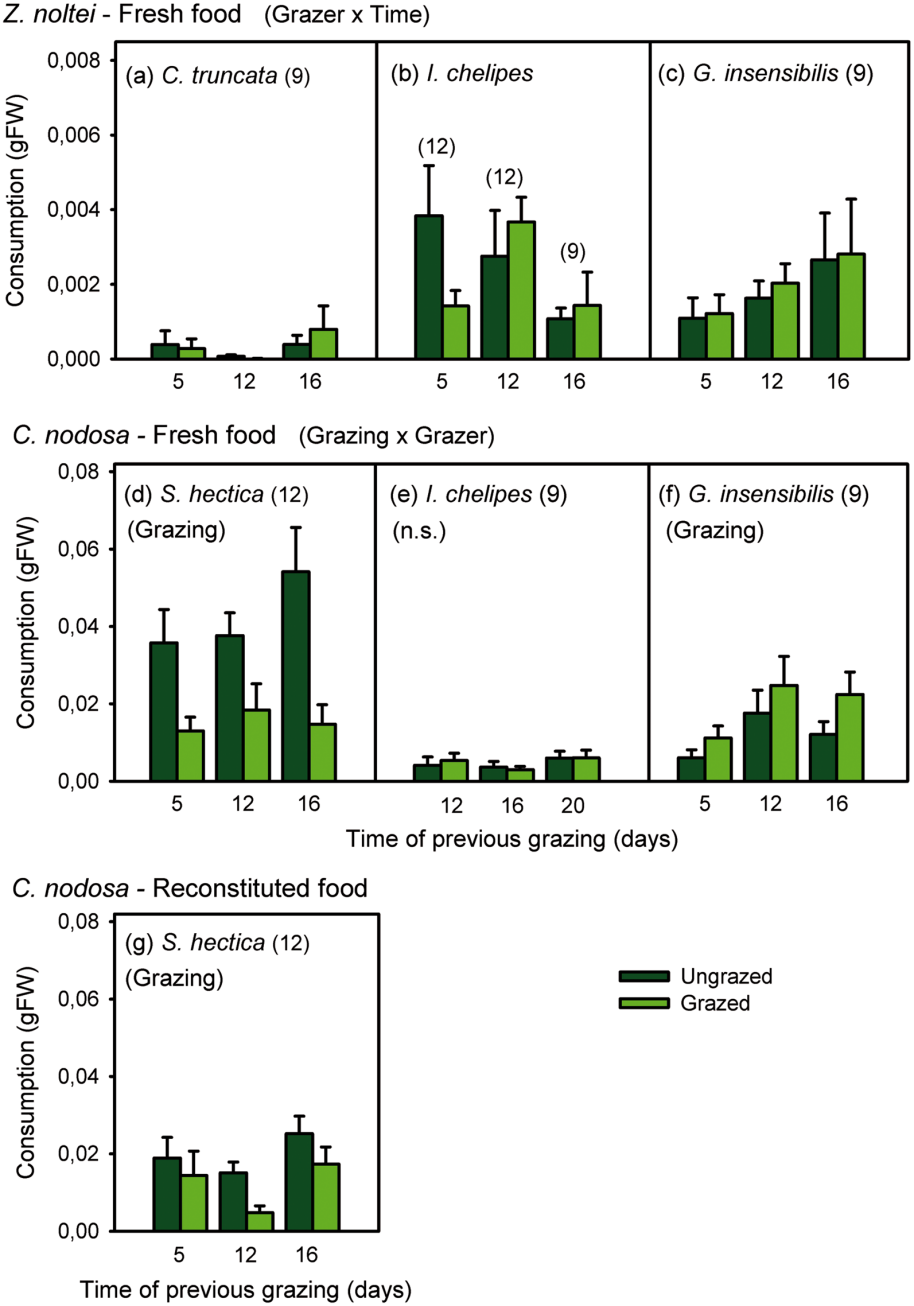
Results of feeding assays examining the effect of previous grazing on seagrass consumption. Mean consumption (± SE) of previously grazed and ungrazed blades of fresh *Zostera noltei* (a-c) and *Cymodocea nodosa* (d-f) after 5, 12, and 16 or 20 days of previous grazing by different mesograzer species, as well of agar-reconstituted food when significant preferences indicating induction were detected with fresh blades (g). Significant interaction for each seagrass species separately is shown in parentheses following the seagrass name. Significant effect of previous grazing on consumption for each grazer separately is shown in parentheses within each graph (n.s. = non-significant). Numbers in parentheses indicate sample size. FW = fresh weight.

Overall consumption of *Z*. *noltei* by *I*. *chelipes* and *G*. *insensibilis* was significantly higher, most of the time, than by *C*. *truncata* (significant Grazer x Time interaction in Fig 5a–5c and S4 Table). No significant differences in the consumption of previously grazed and ungrazed *Z*. *noltei* blades were detected for any mesograzer species at any time (Fig 5a–5c).

Changes in the palatability of previously grazed *C*. *nodosa* shoots significantly influenced the feeding preferences of two grazer species, but in contrasting ways. Previously ungrazed *C*. *nodosa* blades were significantly preferred by the isopod *S*. *hectica* over blades that were previously grazed for 5, 12, and 16 days (Fig 5d and S4 Table). The preference of *S*. *hectica* towards ungrazed blades was maintained when agar-reconstituted food was offered, i.e. when structural seagrass traits were eliminated (Fig 5g), but showing a significant reduction in size compared to fresh blades (0.03 ± 0.01 and 0.01 ± 0.001 effect size for fresh and reconstituted food, respectively; Mann-Whitney U = 340, p < 0.001). In contrast, the amphipod *G*. *insensibilis* significantly preferred previously grazed blades at any time (Fig 5f). *I*. *chelipes* showed no significant preference and consumed similar amounts of ungrazed blades and of those previously grazed for 12, 16, or 20 days (Fig 5e).

Average estimation of grazing intensity obtained from overall consumption rates in feeding assays showed that *S*. *hectica* consumed the highest proportion of the shoot-specific *C*. *nodosa* biomass (23.1%), which was twice as much as the proportion of *Z*. *noltei* biomass consumed by *I*. *chelipes* (10.2%) and *G*. *insensibilis* (9.4%) and the proportion of *C*. *nodosa* consumed by *G*. *insensibilis* (10.5%). The lowest proportions were consumed by *C*. *truncata* on *Z*. *noltei* (1.3%) and by *I*. *chelipes* on *C*. *nodosa* (3.0%). The estimated *S*. *hectica* grazing intensity was similar to the proportion of biomass loss measured in the *C*. *nodosa* growth induction experiment (25.9%, Fig 4d), while the estimated *I*. *chelipes* grazing intensity on *Z*. *noltei* was higher than the value measured when considering the leaf growth (-2.6%; Fig 4b).

### Seagrass chemical traits

*C*. *nodosa* blades that were grazed by *S*. *hectica* showed a significantly higher total phenolic content than ungrazed blades. No significant differences were detected in total nitrogen content or in C:N ratio between ungrazed and grazed blades (Table 1).

**Table 1:** Chemical traits of *Cymodocea nodosa* blades previously ungrazed and grazed by the isopod *Synischia hectica*.

	Ungrazed	Grazed
Nitrogen (% DW)	2.16 (± 0.01)	2.10 (± 0.01)
C:N ratio	19.3 (± 0.2)	19.9 (± 0.1)
Total phenolics (% DW)	3.1 (± 0.1)*	4.5 (± 0.2)*

Values are mean ± SE (n = 2). Asterisks denote significant differences (Welch´s t test, p < 0.05). DW = dry weight.

## Discussion

To our knowledge, this study provides the first report of induction of anti-herbivory defences in seagrasses that deter further consumption. We found that the feeding by the isopod *S*. *hectica* was consistently deterred by the changes that this grazer induced in the seagrass *C*. *nodosa*, as reflected by the significantly lower consumption of blades previously grazed for 5, 12, and 16 days than of previously ungrazed blades. The consistent lower consumption of grazed tissues when offered fresh blades as well as agar-reconstituted food in which seagrass structural traits were eliminated, suggests that chemical traits mediated the isopod deterrence of grazed *C*. *nodosa*. Removal of plant structure, however, did reduce the size of the previous grazing effect, indicating that chemical as well as structural mechanisms of defence coexist. This is in agreement with previous studies in terrestrial plants, freshwater macrophytes, and seaweeds, which showed that multiple chemical and structural traits with additive or even synergistic effect are usually involved in plant defence against specific grazers [45, 47, 48]. The increase in total phenolics but no significant change in C:N ratio and nitrogen content of grazed *C*. *nodosa* blades points out an induction of secondary metabolites rather than a modification of nutritional quality as primary grazer-induced chemical traits mediating the deterrent effect. Interestingly, reports of changes in total phenolic content in response to simulated or direct grazing are not uniform in seagrasses, indicating either a decrease [30] or an increase [29, 31], but showing no subsequent deterrent effect on grazer consumption. Furthermore, previous studies in terrestrial plants and seaweeds showed that the grazer-specific anti-herbivory activity of phenolics is linked to specific compounds or a group of compounds rather than to total phenolics [49, 50]. We acknowledge that specific anti-herbivore compounds that have not yet been identified in seagrasses, as well as other undetermined traits related to leaf nutritional quality and chemistry (e.g. proteins, sugars), could have changed in response to grazing and may have also contributed to the lower consumption of previously grazed *C*. *nodosa*.

We observed contrasting responses to grazing between seagrass species. While the seagrass *C*. *nodosa* induced anti-herbivory defences in response to *S*. *hectica* grazing, *Z*. *noltei* did not induce such defences against herbivory by any of the selected mesograzer species. A likely explanation behind the observed lack of response is that some seagrass species may possess constitutive defences against consumers [51], and plants constitutively defended might not need inducible defences. According to the ODT, slower growing plants are predicted to invest more heavily in constitutive rather than induced defences [1], while a stronger investment in induced defences is predicted by fast-growing plants because resource allocation to growth may limit their investment on constitutive defences [5]. We found, a faster growth rate of *C*. *nodosa* that was closely related to its higher shoot size (i.e. similar shoot growth rates relative to biomass). However, the hypothesis that *Z*. *noltei* could be better constitutively defended than *C*. *nodosa* linked to its lower growth rate was not supported by our results, since *I*. *chelipes* consumed at least as much *Z*. *noltei* as undefended *C*. *nodosa* (i.e. ungrazed *C*. *nodosa* blades). This lack of relationship between growth rate and defences contrasts with ODT predictions, but it is in line with a recent study in terrestrial plants that found that plant competitive ability rather than growth rate is the explanation behind the tradeoff between constitutive and induced defences [52]. In contrast to this hypothesis, we found that *Z*. *noltei* was able to counteract grazing losses to *I*. *chelipes* via compensatory growth, probably due to the moderate biomass consumed by this isopod even when it fed preferentially in young blades. Our observations support ODT predictions of fitness maximization by different defensive patterns depending on the grazing impact, while provide a first hint to a differential role of resistance (i.e. inducible defences) and tolerance mechanisms (i.e. compensatory growth) against herbivory in seagrasses.

Our results revealed the important role of grazer identity in the induction of defences by seagrasses, which is in agreement with previous studies on both terrestrial plants (e.g. [53]) and seaweeds (e.g. [54]). Of the three selected mesograzers, only *S*. *hectica* induced anti-herbivory defences on *C*. *nodosa*. This mesograzer was the largest examined and exerted the strongest grazing impact not only on overall consumption and grazing over time but also by consuming younger *C*. *nodosa* blades. Young leaves are metabolically and photosynthetically more active than older and senescent ones and contain the leaf meristems [55, 56], as such they are closely coupled with plant fitness [7, 57]. Consequently, by preferentially and intensively feeding on younger seagrass blades, *S*. *hectica* is expected to have a large impact on seagrass fitness, which may justify the plant investment in plastic defences. Following the same reasoning, the low grazing intensity and the selective feeding on older tissues by *G*. *insensibilis* and *I*. *chelipes* may at least partially explain the lack of induced defences. We found that the strong grazing impact by *S*. *hectica* exceeded potential tolerance mechanism such as compensatory growth of grazed shoots, as reflected by their significant decrease in biomass during the induction phase. These results are in agreement with a previous study that found compensatory growth of *C*. *nodosa* in response to simulated herbivory of low intensity but not of high intensity [58]. Overall, our results suggest that the intensity of grazing impact in terms of amount of loss of valuable tissues triggered the induction of defences in *C*. *nodosa*. Induction dependence on grazing intensity is in agreement with previous studies in terrestrial systems [59, 60] and seaweeds [61]. The continuous induction of defences observed in our study from 5 to 16 days of previous grazing contrasts with the temporal variation of grazer-deterrent responses found by previous studies in seaweeds [2, 62].

In contrast to the isopod *S*. *hectica*, the amphipod *G*. *insensibilis* preferred previously grazed *C*. *nodosa* blades. This amphipod preferentially fed on old and senescent blades, which are usually less nutritious and chemically defended while accumulated more epibionts compared to young tissues [63]. *G*. *insensibilis* is probably more attracted by the *C*. *nodosa* epibionts than by the seagrass itself, which may benefit seagrass well-being by reducing epiphyte loads [64, 65]. At the same time, previous work on seaweeds might provide an alternative explanation in which habitat and food choice for marine amphipods is driven by the minimization of predation risk rather than by the food chemical defences [66, 67]. In this choice, traits associated with phylogenetic adaptations (tolerance, body size or morphological adaptations) rather than with more plastic feeding behaviour can be at play [68].

## Conclusions

We presented here the first experimental evidence of seagrass induction of anti-herbivory defences that deter further consumption in response to direct grazing by mesograzers. These inducible defences involved a combination of chemical and structural traits. The deployment of this phenotypic plasticity was dependent on the identity of the species involved in the interaction, and particularly on the mesograzer-specific grazing impact. *C*. *nodosa* responded to a high grazing intensity on younger blades by deterring the attacking mesograzers on demand, while it tolerated a low grazing on older blades without inducing defences. Furthermore, *Z*. *noltei* compensated a moderate loss of young biomass by increasing aboveground growth of damaged shoots with no measurable investment in the production of anti-herbivory defences. Overall, our results reinforce previous findings of terrestrial studies showing that different herbivores may cause different grazing damage of different intensity being associated with complementary plant mechanisms of tolerance and resistance [69].

## Supporting Information

S1 Table Results of the RM-ANOVAs examining the effect of time on the proportion of grazed blades during the induction phase. Time = within-subject measure (four levels). Data for each seagrass species were analysed separately. Tests considered: (a) the different grazer species (between-subject factor, three levels; two-way RM-ANOVAs) and (b) each grazer species separately (one-way RM-ANOVAs). (c) Paired t tests used as post-hoc tests when a significant Time effect was detected.
(DOC)

S2 Table Results of the RM-ANOVAs examining the effect of the age of the seagrass blades on the number of bite marks during the induction phase. Blade age = within-subject measure (three levels: young, intermediate, and old). Data for each seagrass species were analysed separately. Tests considered: (a) the different grazer species (between-subject factor, three levels; two-way RM-ANOVAs) and (b) each grazer species separately (one-way RM-ANOVAs). (c) Paired t tests (or Wilcoxon signed-rank tests when data did not meet normality*) used as post-hoc tests when a significant Age effect was detected.
(DOC)

S3 Table Results of the unpaired t tests examining the effect of grazing on seagrass growth rate (g FW shoot^-1^ day^-1^) and biomass (g DW shoot^-1^) during the induction phase. *Z*. *noltei* was exposed to grazing by *I*. *chelipes* and *C*. *nodosa* to grazing by *S*. *hectica* (n = 12).
(DOC)

S4 Table Results of the RM-ANOVAs examining the effect of previous grazing on seagrass consumption by different mesograzer species in feeding assays at different times of previous grazing. Previous grazing = within-subject measure (two levels: grazed and ungrazed) and Time = between-subject measures (three levels). Data for each seagrass species were analysed separately. Tests considered: (a) the different grazer species as between-subject factor with three levels in three-way RM-ANOVAs and (b) each grazer species separately when a significant interaction was detected (two-way RM-ANOVAs).
(DOC)
