# Supplementary material for: Specificity in Mesograzer-Induced Defences in Seagrasses
Source: PLoS One. 2015 Oct 27;10(10):e0141219. doi: 10.1371/journal.pone.0141219 (PMC4624237; doi:10.1371/journal.pone.0141219)
Supplement: S1 Table — Time = within-subject measure (four levels). Data for each seagrass species were analysed separately. Tests considered: (a) the different grazer species (between-subject factor, three levels; two-way RM-ANOVAs) and (b) each grazer species separately (one-way RM-ANOVAs). (c) Paired t tests used as post-hoc tests when a significant Time effect was detected. (DOC) [file pone.0141219.s001.doc]

**Table S1.** **Results of the RM-ANOVAs examining the effect of time on the proportion of grazed blades during the induction phase.** Time = within-subject measure (four levels). Data for each seagrass species were analysed separately. Tests considered: (a) the different grazer species (between-subject factor, three levels; two-way RM-ANOVAs) and (b) each grazer species separately (one-way RM-ANOVAs). (c) Paired t tests used as post-hoc tests when a significant Time effect was detected.

|  |  | Factor | MS | SS | df | F | p-level |
| --- | --- | --- | --- | --- | --- | --- | --- |
| (a) two-way RM-ANOVAs | *Z. noltei* * | Time | 4897 | 10461 | 2.1 | 28 | <0.00001 |
|  |  | Grazer | 17539 | 35078 | 2.0 | 12 | 0.0002 |
|  |  | Time x Grazer | 2057 | 8786 | 4.3 | 12 | <0.00001 |
|  |  | Error(Time) | 173 | 9982 | 58 |  |  |
|  | *C. nodosa* * | Time | 2361 | 3643 | 1.5 | 19 | <0.00001 |
|  |  | Grazer | 32813 | 65626 | 2.0 | 137 | <0.00001 |
|  |  | Time x Grazer | 1105 | 3410 | 3.1 | 9 | 0.0001 |
|  |  | Error(Time) | 126 | 5244 | 42 |  |  |
| (b) one-way RM-ANOVAs | *Z. noltei* - *C. truncata* * | Time | 56 | 69 | 1.2 | 1.1 | 0.325 |
|  |  | Error | 49 | 483 | 9.9 |  |  |
|  | *Z. noltei* - *I. chelipes* | Time | 6165 | 18496 | 3.0 | 24 | <0.00001 |
|  |  | Error | 253 | 8357 | 33 |  |  |
|  | *Z. noltei* - *G. insensibilis* * | Time | 2677 | 3299 | 1.2 | 23 | 0.0005 |
|  |  | Error | 116 | 1143 | 9.9 |  |  |
|  | *C. nodosa* - *S. hectica* * | Time | 6500 | 7276 | 1.1 | 23 | 0.0003 |
|  |  | Error | 279 | 3434 | 12 |  |  |
|  | *C. nodosa* - *I. chelipes* | Time | 1.1 | 3.3 | 3.0 | 0.2 | 0.903 |
|  |  | Error | 5.9 | 142 | 24 |  |  |
|  | *C. nodosa* - *G. insensibilis* * | Time | 620 | 785 | 1.3 | 3.8 | 0.074 |
|  |  | Error | 165 | 1669 | 10 |  |  |

* Data that do not meet sphericity for which corrected degrees of freedom from Greenhouse–Geisser adjustment were used.

| (c) one-way RM-ANOVA post hoc comparisons |  | t | df | p-level |
| --- | --- | --- | --- | --- |
| *Z. noltei* - *I. chelipes* | Day3 - Day6 | -5.7 | 11 | 0.0001 |
|  | Day3 - Day10 | -5.9 | 11 | 0.0001 |
|  | Day3 - Day12 | -7.4 | 11 | 0.00001 |
|  | Day6 - Day10 | -2.8 | 11 | 0.019 |
|  | Day6 - Day12 | -3.3 | 11 | 0.007 |
|  | Day10 - Day12 | -1.4 | 11 | 0.182 |
| *Z. noltei* - *G. Insensibilis* | Day3 - Day6 | -4.9 | 8 | 0.001 |
|  | Day3 - Day10 | -4.8 | 8 | 0.001 |
|  | Day3 - Day12 | -5.2 | 8 | 0.001 |
|  | Day6 - Day10 | -4.3 | 8 | 0.003 |
|  | Day6 - Day12 | -4.5 | 8 | 0.002 |
|  | Day10 - Day12 | -2.4 | 8 | 0.046 |
| *C. nodosa* - *S. hectica* | Day3 - Day6 | -3.5 | 11 | 0.005 |
|  | Day3 - Day10 | -4.9 | 11 | 0.0005 |
|  | Day3 - Day12 | -6.2 | 11 | 0.0001 |
|  | Day6 - Day10 | -17.2 | 11 | <0.00001 |
|  | Day6 - Day12 | -5.5 | 11 | 0.0002 |
|  | Day10 - Day12 | -0.5 | 11 | 0.604 |
