# Supplementary material for: Specificity in Mesograzer-Induced Defences in Seagrasses
Source: PLoS One. 2015 Oct 27;10(10):e0141219. doi: 10.1371/journal.pone.0141219 (PMC4624237; doi:10.1371/journal.pone.0141219)
Supplement: S2 Table — Blade age = within-subject measure (three levels: young, intermediate, and old). Data for each seagrass species were analysed separately. Tests considered: (a) the different grazer species (between-subject factor, three levels; two-way RM-ANOVAs) and (b) each grazer species separately (one-way RM-ANOVAs). (c) Paired t tests (or Wilcoxon signed-rank tests when data did not meet normality*) used as post-hoc tests when a significant Age effect was detected. (DOC) [file pone.0141219.s002.doc]

**Table S2.** **Results of the RM-ANOVAs examining the effect of the age of the seagrass blades on the number of bite marks during the induction phase.** Blade age = within-subject measure (three levels: young, intermediate, and old). Data for each seagrass species were analysed separately. Tests considered: (a) the different grazer species (between-subject factor, three levels; two-way RM-ANOVAs) and (b) each grazer species separately (one-way RM-ANOVAs). (c) Paired t tests (or Wilcoxon signed-rank tests when data did not meet normality*) used as post-hoc tests when a significant Age effect was detected.

|  |  | Factor | MS | SS | df | F | p-level |
| --- | --- | --- | --- | --- | --- | --- | --- |
| (a) two-way RM-ANOVAs | *Z. noltei* * | Age | 2.0 | 3.3 | 1.6 | 14 | 0.00004 |
|  |  | Grazer | 6.9 | 14 | 2 | 29 | <0.00001 |
|  |  | Age x Grazer | 0.5 | 1.6 | 3.3 | 3.4 | 0.02 |
|  |  | Error(Age) | 0.1 | 6.2 | 45 |  |  |
|  | *C. nodosa* * | Age | 0.6 | 0.8 | 1.5 | 5.6 | 0.01 |
|  |  | Grazer | 7.8 | 16 | 2.0 | 43 | <0.00001 |
|  |  | Age x Grazer | 5.0 | 15 | 3.0 | 50 | <0.00001 |
|  |  | Error(Age) | 0.1 | 4.0 | 40 |  |  |
| (b) one-way RM-ANOVAs | *Z. noltei* - *C. truncata* | Age | 0.02 | 0.03 | 2 | 3.4 | 0.060 |
|  |  | Error | 0.005 | 0.1 | 16 |  |  |
|  | *Z. noltei* - *I. chelipe*s | Age | 1.9 | 3.9 | 2 | 10 | 0.001 |
|  |  | Error | 0.2 | 4.3 | 22 |  |  |
|  | *Z. noltei* - *G. insensibilis* * | Age | 1.4 | 1.5 | 1.1 | 6.4 | 0.032 |
|  |  | Error | 0.2 | 1.9 | 8.6 |  |  |
|  | *C. nodosa* - *S. hectica* * | Age | 11 | 15 | 1.4 | 55 | <0.00001 |
|  |  | Error | 0.2 | 3.0 | 15 |  |  |
|  | *C. nodosa* - *I. chelipes* | Age | 0.2 | 0.4 | 2 | 7.9 | 0.004 |
|  |  | Error | 0.03 | 0.4 | 16 |  |  |
|  | *C. nodosa* - *G. Insensibilis* * | Age | 1.2 | 1.5 | 1.2 | 21 | 0.001 |
|  |  | Error | 0.1 | 0.6 | 10 |  |  |

* Data that do not meet sphericity for which corrected degrees of freedom from Greenhouse–Geisser adjustment were used.

| (c) one-way RM-ANOVA post hoc comparisons |  | t | n | p-level |
| --- | --- | --- | --- | --- |
| *Z. noltei* - *I. chelipes* | Y-I | 0.1 | 12 | 0.921 |
|  | Y-O* | - | 12 | 0.007 |
|  | I-O* | - | 12 | 0.006 |
| *Z. noltei* - *G. Insensibilis* | Y-I | -1.2 | 9 | 0.27 |
|  | Y-O | 2.0 | 9 | 0.08 |
|  | I-O | 7.7 | 9 | 0.0001 |
| *C. nodosa* - *S. hectica* | Y-I | 4.3 | 12 | 0.001 |
|  | Y-O* | - | 12 | 0.002 |
|  | I-O* | - | 12 | 0.002 |
| *C. nodosa* - *I. chelipes* | Y-I* | - | 9 | 0.16 |
|  | Y-O* | - | 9 | 0.017 |
|  | I-O* | - | 9 | 0.042 |
| *C. nodosa* - *G. Insensibilis* | Y-I* | - | 9 | 0.042 |
|  | Y-O* | - | 9 | 0.007 |
|  | I-O | -6.9 | 9 | 0.0001 |
