# Supplementary material for: Specificity in Mesograzer-Induced Defences in Seagrasses
Source: PLoS One. 2015 Oct 27;10(10):e0141219. doi: 10.1371/journal.pone.0141219 (PMC4624237; doi:10.1371/journal.pone.0141219)
Supplement: S3 Table — Z. noltei was exposed to grazing by I. chelipes and C. nodosa to grazing by S. hectica (n = 12). (DOC) [file pone.0141219.s003.doc]

**Table S3.** **Results of the unpaired t tests examining the effect of grazing on seagrass growth rate (g FW shoot^-1^ day^-1^) and biomass (g DW shoot^-1^) during the induction phase.** *Z. noltei* was exposed to grazing by *I. chelipes* and *C. nodosa* to grazing by *S. hectica* (n = 12).

|  |  | t | df | p-level |
| --- | --- | --- | --- | --- |
| *Z. noltei* - *I. chelipes* | Growth rate | -2.2 | 22 | 0.042 |
|  | Biomass | -0.3 | 22 | 0.772 |
| *C. nodosa* - *S. hectica* | Growth rate | 0.8 | 22 | 0.407 |
|  | Biomass | 3.1 | 22 | 0.006 |
