# Supplementary material for: Specificity in Mesograzer-Induced Defences in Seagrasses
Source: PLoS One. 2015 Oct 27;10(10):e0141219. doi: 10.1371/journal.pone.0141219 (PMC4624237; doi:10.1371/journal.pone.0141219)
Supplement: S4 Table — Previous grazing = within-subject measure (two levels: grazed and ungrazed) and Time = between-subject measures (three levels). Data for each seagrass species were analysed separately. Tests considered: (a) the different grazer species as between-subject factor with three levels in three-way RM-ANOVAs and (b) each grazer species separately when a significant interaction was detected (two-way RM-ANOVAs). (DOC) [file pone.0141219.s004.doc]

**Table S4.** **Results of the RM-ANOVAs examining the effect of previous grazing on seagrass consumption by different mesograzer species in feeding assays at different times of previous grazing**. Previous grazing = within-subject measure (two levels: grazed and ungrazed) and Time = between-subject measures (three levels). Data for each seagrass species were analysed separately. Tests considered: (a) the different grazer species as between-subject factor with three levels in three-way RM-ANOVAs and (b) each grazer species separately when a significant interaction was detected (two-way RM-ANOVAs).

|  |  | Factor | MS | df | F | p-level |
| --- | --- | --- | --- | --- | --- | --- |
| (a) three-way RM-ANOVAs | *Z. noltei* | Grazing | 0.00000003 | 1 | 0.004 | 0.95 |
|  |  | Grazer | 0.0001 | 2 | 11 | 0.0001 |
|  |  | Time | 0.000002 | 2 | 0.3 | 0.77 |
|  |  | Grazer x Time | 0.00002 | 4 | 2.6 | 0.04 |
|  |  | Grazing x Grazer | 0.000002 | 2 | 0.2 | 0.80 |
|  |  | Grazing x Time | 0.00001 | 2 | 1.0 | 0.38 |
|  |  | Grazing x Grazer x Time | 0.00001 | 4 | 0.8 | 0.55 |
|  |  | Error(Grazing) | 0.00001 | 78 |  |  |
|  | *C. nodosa* | Grazing | 0.002 | 1 | 5.1 | 0.026 |
|  |  | Grazer | 0.01 | 2 | 29 | <0.00001 |
|  |  | Time | 0.001 | 2 | 2.2 | 0.117 |
|  |  | Grazer x Time | 0.0003 | 4 | 1.0 | 0.417 |
|  |  | Grazing x Grazer | 0.01 | 2 | 15 | <0.00001 |
|  |  | Grazing x Time | 0.0001 | 2 | 0.3 | 0.72 |
|  |  | Grazing x Grazer x Time | 0.0003 | 4 | 0.7 | 0.57 |
|  |  | Error(Grazing) | 0.0004 | 81 |  |  |
| (b) two-way RM-ANOVAs | *C. nodosa*-*S. hectica* | Grazing | 0.01 | 1 | 18 | 0.0002 |
|  |  | Time | 0.001 | 2 | 1.1 | 0.33 |
|  |  | Grazing x Time | 0.001 | 2 | 0.9 | 0.40 |
|  |  | Error(Grazing) | 0.001 | 33 |  |  |
|  | *C. nodosa*-*I. chelipes* | Grazing | 0.0000005 | 1 | 0.02 | 0.89 |
|  |  | Time | 0.00003 | 2 | 1.0 | 0.39 |
|  |  | Grazing x Time | 0.000004 | 2 | 0.2 | 0.85 |
|  |  | Error(Grazing) | 0.00002 | 24 |  |  |
|  | *C. nodosa*-*G. insensibilis* | Grazing | 0.001 | 1 | 4.7 | 0.04 |
|  |  | Time | 0.001 | 2 | 2.6 | 0.10 |
|  |  | Grazing x Time | 0.00003 | 2 | 0.2 | 0.83 |
|  |  | Error(Grazing) | 0.0002 | 24 |  |  |
|  | Agar-based  *C. nodosa*-*S. hectica* | Grazing | 0.001 | 1 | 46 | <0.00001 |
|  |  | Time | 0.001 | 2 | 1.7 | 0.20 |
|  |  | Grazing x Time | 0.0001 | 2 | 2.3 | 0.12 |
|  |  | Error(Grazing) | 0.00002 | 33 |  |  |
